# Pd_3_Co_1_ Alloy Nanocluster on the MWCNT Catalyst for Efficient Formic Acid Electro-Oxidation

**DOI:** 10.3390/nano12234182

**Published:** 2022-11-25

**Authors:** Pingping Yang, Li Zhang, Xuejiao Wei, Shiming Dong, Yuejun Ouyang

**Affiliations:** College of Chemistry and Materials Engineering, Huaihua University, Huaihua 418008, China

**Keywords:** Pd_3_Co_1_ alloy nanocluster, multiwalled carbon nanotubes, formic acid oxidation, deep eutectic solvents, direct formic acid fuel cell

## Abstract

In this study, the Pd_3_Co_1_ alloy nanocluster from a multiwalled carbon nanotube (MWCTN) catalyst was fabricated in deep eutectic solvents (DESs) (referred to Pd_3_Co_1_/CNTs). The catalyst shows a better mass activity towards the formic acid oxidation reaction (FAOR) (2410.1 mA mg_Pd_^−1^), a better anti-CO toxicity (0.36 V) than Pd/CNTs and commercial Pd/C. The improved performance of Pd_3_Co_1_/CNTs is attributed to appropriate Co doping, which changed the electronic state around the Pd atom, lowered the d-band of Pd, formed a new Pd-Co bond act at the active sites, affected the adsorption of the toxic intermediates and weakened the dissolution of Pd; moreover, with the assistance of DES, the obtained ultrafine Pd_3_Co_1_ nanoalloy exposes more active sites to enhance the dehydrogenation process of the FAOR. The study shows a new way to construct a high-performance Pd-alloy catalyst for the direct formic acid fuel cell.

## 1. Introduction

The development of fuel cells is one of the important ways to achieve carbon neutrality [1,2,3,4,5]. A direct formic acid fuel cell (DFAFC), with the following advantages, a high theoretical energy density, a low operating temperature and easy management, are attracting a lot of interest [6,7,8,9,10].

Palladium (Pd) has a good catalytic performance for DFAFC [11,12,13,14]. Various Pd-based bimetallic alloy catalysts (e.g., PdCu [15], PdMn [16], PdW [17], PdCO [18], PdIr [19], PdSn [20], PdNi [21]) have been prepared to improve the inert FAOR because these alloys exhibited the ability to get rid of OH species. Among them, the PdCo-alloys have attracted great attention. Zhang et al. fabricated a PdCo Nds-RGO catalyst exhibiting the excellent peak current density toward the FAOR [22]. Li et al. prepared a PdCo_0.70_/C catalyst showing an enhanced mass activity toward the FAOR [23]. Hence, PdCo-based catalysts have the great potential in a DFAFC. However, the instability of the catalysts caused by the dissolution of metal, the Ostwald ripening, and the corrosion of the carbon carrier have not been solved [22,24,25]. Therefore, how to improve the electrochemical catalytic activity and stability of the PdCo-based catalysts are the current focus of researchers’ efforts.

Doping [17,26,27,28] is considered to be the most effective method to improve the stability and activity of Pd-alloy catalysts because the doped metal can change the electronic state around the Pd atom, affecting the adsorption of the toxic intermediates and weakening the dissolution of Pd. Tang et al. doped Co into Pd to form an alloy, which significantly improved the activity and stability of the catalyst [29]. In addition, Pd can also form alloy catalysts with other metals (Cu, Ni, Ru) by doping, which is characterized by the improved intrinsic activity and stability toward the FAOR [30,31,32].

It is well known that increasing the loading rate of metal nanoparticles on a support is an effective method to improve the activity of the catalysts. With these advantages, a good electrical conductivity, thermal stability, electrochemical stability, a negligible vapor pressure, non-toxicity and biodegradability, DES [33,34,35] was used as a green solvent to prepare catalysts. In our previous study, a series of Pt- and Pd-based high-performance catalysts were prepared using DES as the reaction solvent [36,37,38,39,40]. Fan et al. prepared the alloy catalysts (PdSn [20], PtCo [34], PtCu [33], PtV [41], PtLa [42]) by a chemical reduction or electrochemistry method in DES, that plays an important role in controlling the shape of these nanocrystallines. These catalysts showed an excellent mass activity in the fuel cell. Zhuo et al. electrochemically synthesized the AuPt nanopowers in DES and applied them for the organic electrooxidation [43]. Wei et al., prepared concave cubic PtSm alloy nanocrystals [44], concave-disdyakis triacontahedral Pd nanocrystals [45] and cubic Pt_93_Ir_7_ [46] with high-index facets that exhibit a higher electrocatalytic activity and stability. Hammons et al. prepared Pd nanoparticles electrodeposited from DES and revealed the interaction between the particles and the solvent, showing that this special solvent can stabilize these electrodeposited Pd nanoparticles [47]. Therefore, the high-performance PdCo catalysts are expected to be prepared in DES. 

**Scheme 1:**
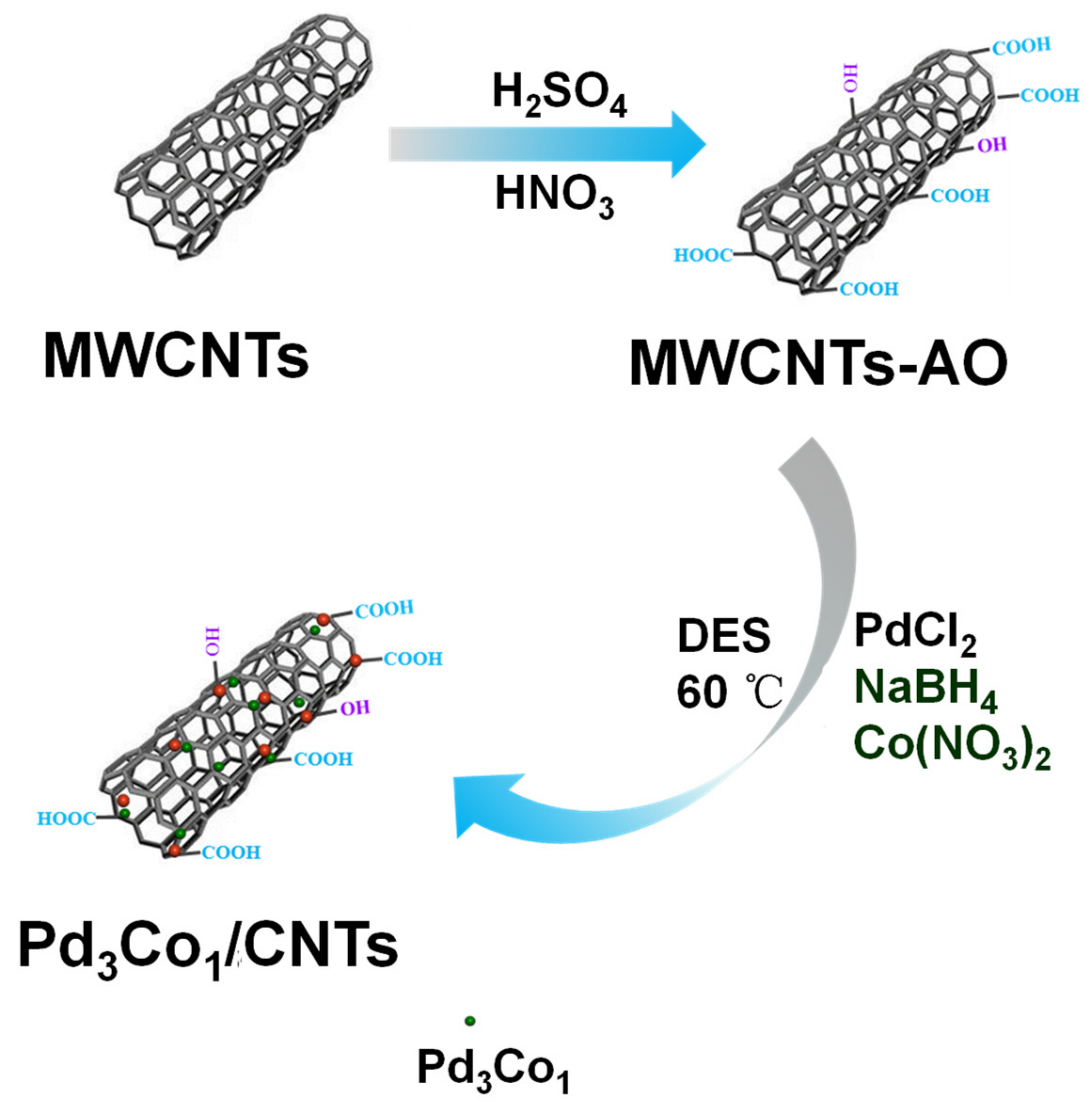
Schematic illustration showing the preparation of Pd_3_Co_1_/CNTs.

MWCNTs, with a unique morphology and excellent properties, have attracted much more interest in a variety of fields [48,49,50,51]. Therefore, in DES, we doped the Co element into Pd to form the PdCo alloy structure and then loaded it on MWCNTs (Pd_3_Co_1_/CNTs) (Scheme 1). The electrochemical tests showed that this catalyst shows an excellent electrocatalytic performance. This study provides a new strategy for the development of a high-performance PdCo alloy FAOR catalyst.

## 2. Materials and Methods

### 2.1. Materials

MWCNTs (OD: 8 nm, Length: 10–30 mm, Purity ¼ 95 wt%) were purchased from the Xianfeng Nanomaterials Technology Co., Ltd., Nanjing, China. Nafion solution (5 wt%) was purchased from Sigma-Aldrich, Saint Louis, MO, USA. Choline chloride [HOC_2_H_4_N(CH_3_)_3_Cl], Urea [CO(NH_2_)_2_], C_2_ H_5_OH, PdCl_2_, Co(NO_3_)_2_, NaBH_4_, H_2_SO_4_, HCOOH and HNO_3_ were purchased from Shanghai Chemical Reagent Co., Ltd., Shanghai, China. All of the reagents used were analytically pure.

### 2.2. Materials Synthesis

Concretely, DES (mole ratio of the urea/choline chloride is 2) was stirred at 80 °C until a homogeneous, colourless liquid developed and was sealed at 60 °C, for later use [37]. All solutions are configured with DES instead of water. Then, the untreated 200 mg MWCNTs were functionalized by an acidification treatment (V_H2SO4_/V_HNO3_ = 3/1) (referred to CNTs-AO) and sealed at room temperature, for later use. A 10 mg CNTs-AO, 0.33 mL PdCl_2_ /DESs solution (10 mg/mL), and 0.11 mL Co(NO_3_)_2_/DESs solution (10 mg/mL) were added in 20 mL DES with an appropriate proportion (mole ratio of Pd/Co is 3). Then, 60 mg NaBH_4_ was added and stirred at 60 °C for three hours at room temperature. The product was washed three times with distilled water and anhydrous ethanol, repeatedly. The material was centrifuged in a centrifugal machine, and dried in a vacuum drying oven at 60 °C all night (referred to Pd_3_Co_1_/CNTs). In the control experiment, the Pd_1_Co_1_/CNT and Pd_1_Co_3_/CNT catalysts were prepared following the same steps, as above, except for the change in the amount of Co(NO_3_)_2_. Pd/CNTs were prepared following the same steps, as above, except for the addition of Co(NO_3_)_2_.

### 2.3. Materials Characterization

The XRD patterns were obtained using an X-ray diffractometer (Rigaku D/MAX 2500 v/pc, Japan) with a Cu K a radiation source (l ¼ 1.5406 Å). The XPS measurements were carried out using a Physical Electronics PHIQuantum 2000 system with an Al K a radiation source, and all of the XPS spectra were calibrated with the C1s line at 284.5 eV. The surface morphologies and the microstructures of the prepared catalysts were analyzed using (SEM, LEO-1530) and (HRTEM, JEOL JEM-2100) with an accelerating voltage of 200 kV. An Agilent 720 inductively coupled plasma-optical emission spectrophotometer (ICP-OES, USA) was used to determine the Pd contents of Pd/C (20%), Pd/CNTs (17.5%), Pd_1_Co_1_/CNTs (18.3%), Pd_1_Co_3_/CNTs (18.6%) and Pd_3_Co_1_/CNTs (19.1%) (Table S1: Supporting Information).

### 2.4. Electrochemical Measurements

According to our previously published protocol, the catalyst-modified glassy carbon electrodes (GC, 5 mm in diameter) were prepared [37]. Using a traditional three-electrode system in an electrochemical workstation (CHI 830b05049, Shanghai Chenhua Instrument Co., Ltd., Shanghai, China) at 25 ℃, all of the electrochemical measurements were performed. The counter electrode and the reference electrode were a platinum foil and a saturated calomel electrode (SCE), respectively. The working electrode was modified as follows: 0.05, 0.3, and 1.0 µm alumina powder was used to polish the GC. With the existence of redistilled water (950 µL) and a Nafion solution (0.5 wt%, 50 μL), the catalysts (2 mg) were dispersed in this solution above (1000 μL). A suspension (10 μL) was trickled over the GC electrode at room temperature. The Pd loading of Pd/C, Pd/CNTs, Pd_1_Co_1_/CNTs, Pd_1_Co_3_/CNTs and Pd_3_Co_1_/CNTs were 23.8, 24.2, 24.3, 24.5, and 24.9 μg cm^−2^.

In a N_2_-saturated 0.5 M H_2_SO_4_ solution, we studied the electrochemical behaviors of all catalysts. The corresponding formic acid electrocatalytic properties of all catalysts were studied in 1.0 M HCOOH + 0.5 M H_2_SO_4_ solution. Furthermore, we used CO stripping experiments to investigate the CO toxicity of all catalysts, in terms of the following steps. Firstly, to remove the dissolved O_2_ in the solution, we degassed the electrolyte by purging with a pure N_2_ airflow for 15 min. Secondly, to allow the saturated adsorption of CO, we degassed the pure CO airflow bubbling for 15 min at a scan rate of 50 mV s^−1^ while maintaining the potential sweep in the range of −0.2 and 0.0 V. Then, to remove the dissolved CO and to avoid the disturbance of O_2_ in the air, we degassed the electrolyte by purging with pure N_2_ for 30 min. Finally, to oxidize the preadsorbed CO on the surface of the catalysts, we executed the CO stripping voltammograms in the range of −0.2 V and 1.0 V. All of the currents in the electrochemical experiments were expressed by the normalized current per milligram of the Pd metal loading on the working electrode. The electrolyte was purged with pure N_2_ for 15 min before each measurement, and impeded the disturbance of O_2_ in the air with a flux of N_2_ flow over the electrolyte.

## 3. Results and Discussion

**Figure 1:**
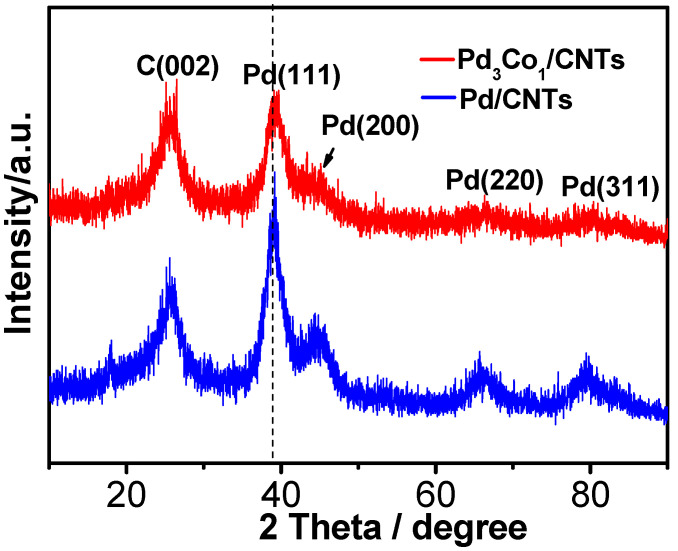
XRD patterns of the Pd/CNT and Pd_3_Co_1_/CNT catalysts.

Figure 1 shows the XRD patterns of the Pd_3_Co_1_/CNT and Pd/CNT catalysts. The 2θ (26.2°) is attributed to C(002) of MWCNTs-AO [37]. The Pd(111), (200), (220) and (311) diffraction peaks of Pd/CNTs at 39.0°, 44.3°, 66.1° and 79.8°, while all corresponding Pd peaks of Pd_3_Co_1_/CNTs shifted to higher 2θ values of 39.2°, 44.6°, 66.3° and 80.1°, suggesting the formation of the Pd_3_Co_1_ alloy [18,52,53]. Meanwhile, (Figure S1: Supporting Information) shows the XRD patterns of the Pd_1_Co_1_/CNT and Pd_1_Co_3_/CNT catalysts. The Pd(111), (200), (220) and (311) diffraction peaks still appear, which indirectly indicates that Co doped into the interior of Pd to form an alloy.

**Figure 2:**
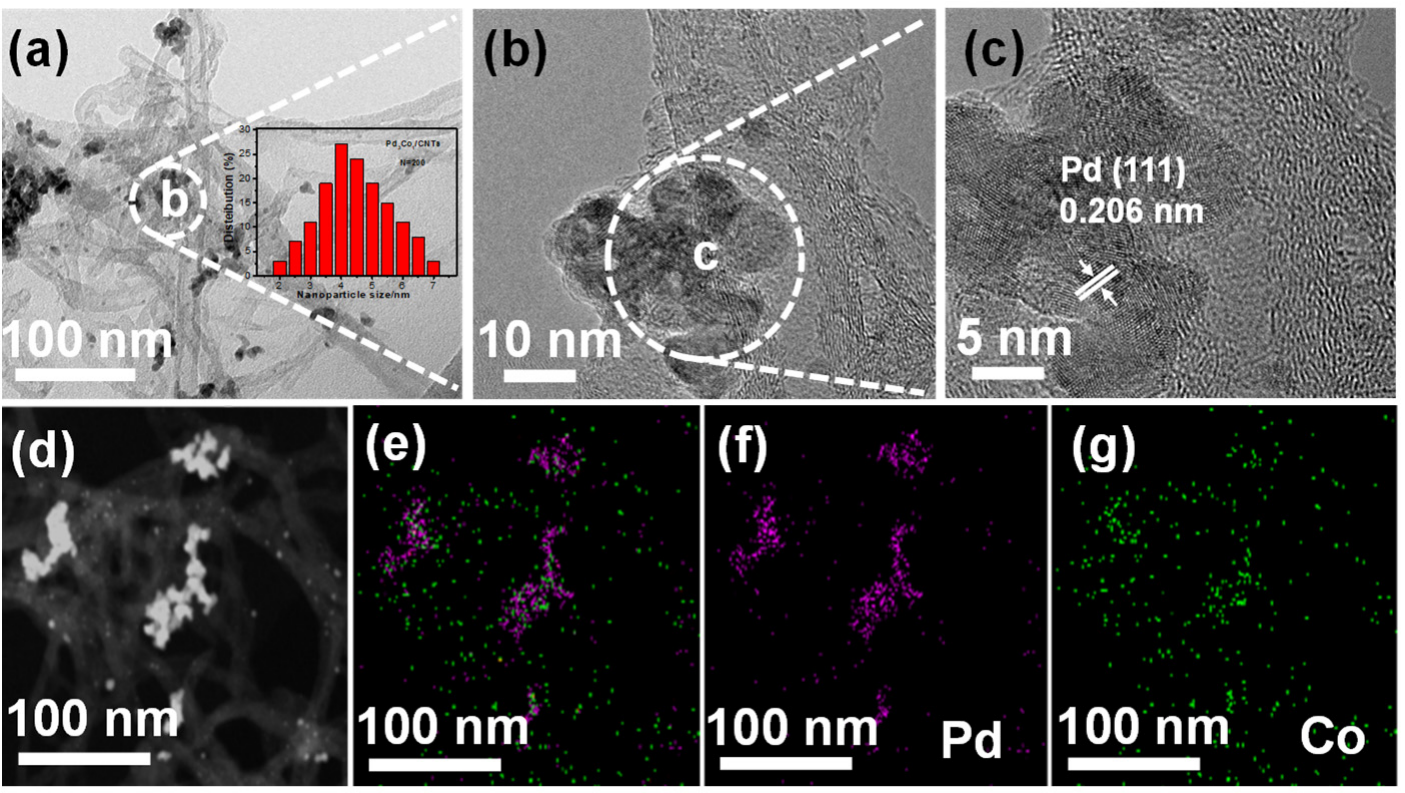
TEM and HRTEM images (the corresponding particle size distribution); HAADF-STEM elements.

Figure 2 shows the TEM (a), HRTEM (b–c) images and the HAADF-STEM element mapping (d–g) results (Pd, Co) and elements of Pd_3_Co_1_/CNTs. Figure 2a,b shows Pd_3_Co_1_ nanoclusters successfully loaded on the surface of MWCNTs. As shown in Figure 2c, the spacings of Pd(111) crystal planes in Pd_3_Co_1_/CNTs to be 0.206 nm, which is different from the Pd(111)(0.222 nm) of Pd/CNTs (Figure S2: Supporting Information). This may be attributed to the doping of Co atoms with a small radius and compressed in the lattice constant of Pd [22]. Figure 2d-g shows that Pd and Co atoms are evenly distributed in the nanoclusters, which also indirectly proves the formation of alloys. In addition, in order to further determine the formation of the alloy structure, the TEM and HRTEM images and the HAADF-STEM element mapping of Pd_3_Co_1_/CNTs were also re-performed in another region, and the results showed that Pd and Co formed an alloy structure (Figure S3: Supporting Information). Figures S4 and S5: Supporting Information show the EDX line-profiles and the spot scanning of the Pd_3_Co_1_ nanoparticle (where Pd is in red and Co in blue), which further proved the existence of the alloy and the molar ratio of Pd and Co is (3.07/1). The average nanoparticles’ sizes of Pd_3_Co_1_/CNTs (Figure 2a), Pd_3_Co_1_/C (Figure S6: Supporting Information), Pd_1_Co_1_/CNTs (Figure S7: Supporting Information), Pd_1_Co_1_/CNTs (Figure S7: Supporting Information) and Pd/CNTs (Figure S8: Supporting Information) are 4.3, 4.5, 4.9, 5.3 and 7.5 nm (200 particles). Furthermore, in order to explore the carrier’s influence on the catalysts, Figure S9: Supporting Information shows the TEM and HRTEM images of Pd_3_Co_1_/C. It can be seen that there is no significant change in the size of the nanoparticles when carbon black is used as the carrier.

**Figure 3:**
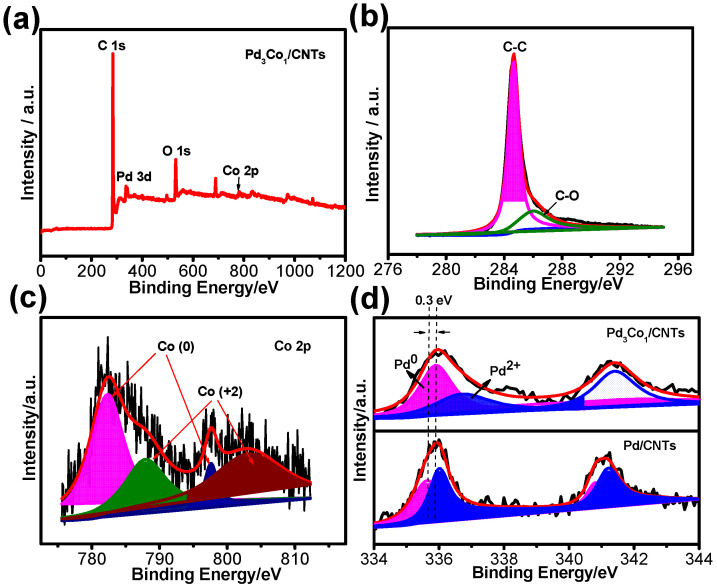
(**a**) XPS survey spectra, (**b**) C (1s) spectrum, (**c**) Co (2p) spectrum of Pd_3_Co_1_/CNTs, (**d**) Pd (3d) spectrum of Pd3Co1/CNTs and Pd/CNTs.

Figure 3a shows the XPS survey spectra of Pd_3_Co_1_/CNTs. The signals corresponding to C 1s (284.6 eV), O 1s (531.2 eV), Pd 3d (338.2 eV), and Co 2p (783.2 eV) are observed. The two splitting peaks of C1s (C-C, C-O) are attributed to MWCNTs-AO (Figure 3b) [38]. Figure 3c shows the peaks of Co (0) and Co (+2), which are the evidence of Co. Figure 3d and Table S2: Supporting Information show a slight positive change (0.3 eV) in the Pd (0) peak position from Pd/CNTs to Pd_3_Co_1_/CNTs, indicating the strong charge transfer interaction between the Co and Pd atoms to form the PdCo bond [54,55], which was the important reason for improving the stability and anti-CO toxicity. Furthermore, the presence of Co (+2) and Pd (+2) is attributed to the oxidation of oxygen in the air [36].

**Figure 4:**
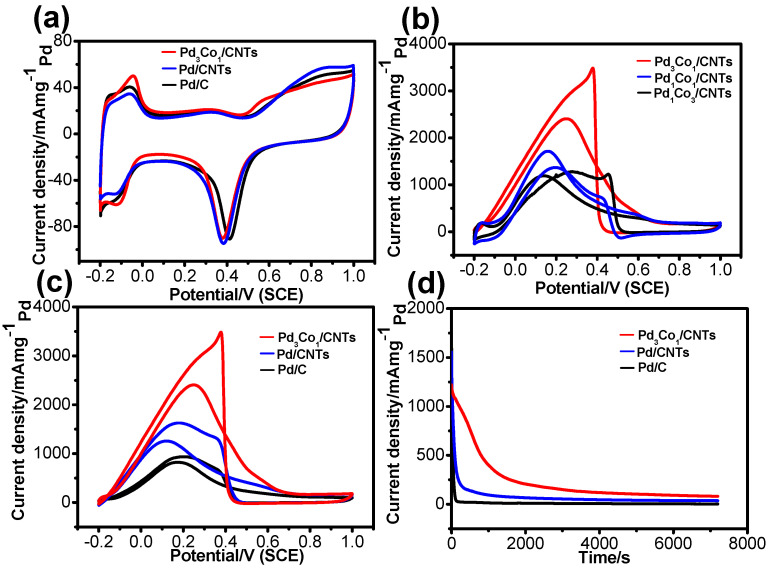
(**a**,**c**) Cyclic voltammograms and (**d**) current-time curves of Pd_3_Co_1_/CNTs, Pd/CNTs and Pd/C; (**b**) cyclic voltammograms curves of Pd_3_Co_1_/CNTs, Pd_1_Co_1_/CNTs and Pd_1_Co_3_/CNTs in 0.5 M H_2_SO_4_/1.0 M HCOOH + 0.5 M H_2_SO_4_ solution.

Figure 4a shows the cyclic voltammograms (CV) of the Pd_3_Co_1_/CNTs, Pd/CNTs and Pd/C catalysts in the 0.5 M H_2_SO_4_ solution. The associated ECSA was calculated by integrating the area associated with the hydrogen adsorption region [56], according to:

ECSA= Q_H_/210 (µC cm^−2^)/W_Pd_

wherein Q_H_ is the integrated charge within the hydrogen adsorption area in the CV curves after subtracting the charge from the double-layer region. It should be noted that the data were obtained from the CV curves with 210 (μC/cm^2^) as the conversion factor. W_Pd_ is the mass of Pd. Therefore, the ECSA of Pd_3_Co_1_/CNTs was calculated to be 41.2 m^2^ g^−1^, which is higher than those of Pd/CNTs (33.4 m^2^ g^−1^), and Pd/C (31.1 m^2^ g^−1^). The larger ECSA of Pd_3_Co_1_/CNTs is attributed to the smaller Pd_3_Co_1_ particles. In order to explore the effect of the atomic ratio on the catalyst performance, we tested the catalytic performance of different Pd/Co catalysts for the FAOR (Figure 4b). In the forward scan, the anodic peak was assigned to the formic acid oxidation, and another anodic peak in the reverse scan was associated with the oxidation of the intermediate carbonaceous species formed during the forward scan [57,58]. For Pd_3_Co_1_/CNTs, the peak current density in the forward scan is 2410.1 mA mg_Pd_^−1^, higher than Pd_1_Co_1_/CNTs (1752.3 mA mg_Pd_^−1^), Pd_1_Co_3_/CNTs (1255.1 mA mg_Pd_^−1^), Pd/CNTs (1262.8 mA mg_Pd_^−1^) and Pd/C (819.2 mA mg_Pd_^−1^) (Figure 4b,c). In order to investigate whether the high activity is achieved by the alloying effect or the supporting effect, we tested the catalytic activity of the carbon-supported Pd_3_Co_1_ alloy catalyst (Pd_3_Co_1_/C) towards the FAOR (Figure S10: Supporting Information). The peak current density of Pd_3_Co_1_/C is 1919.2 mA mg_Pd_^−1^, higher than Pd_1_Co_1_/CNTs (1752.3 mA mg_Pd_^−1^), Pd_1_Co_3_/CNTs (1255.1 mA mg_Pd_^−1^), Pd/CNTs (1262.8 mA mg_Pd_^−1^) and Pd/C (819.2 mA mg_Pd_^−1^), but lower than Pd_3_Co_1_/CNTs (2410.1 mA mg_Pd_^−1^), which revealed that the high activity was achieved through the synergistic effect of the alloy effect and the support effect. The alloy effect is beneficial to enhance the toxicity resistance of the catalyst and the addition of MWCNTs is beneficial to improve the electron transport. To evaluate the long-term performance of all catalysts, the chronoamperometric (CA) measurements were performed at +0.3 V for 7200 s. As shown in Figure 4d, in the initial period, all curves showed a fast current decay that indicated the poisoning of the electrocatalysts, due to the formation of intermediate species [26]. Then, after 7200 s, the Pd_3_Co_1_/CNT catalyst, still maintaining higher current density (95.1 mA mg_Pd_^−1^), was almost 2.6 and 29.7 times greater than Pd/CNTs (36.3 mA mg_Pd_^−1^), and Pd/C (3.2 mA mg_Pd_^−1^), which illustrated that Pd_3_Co_1_/CNTs exhibit a better stability. 

**Figure 5:**
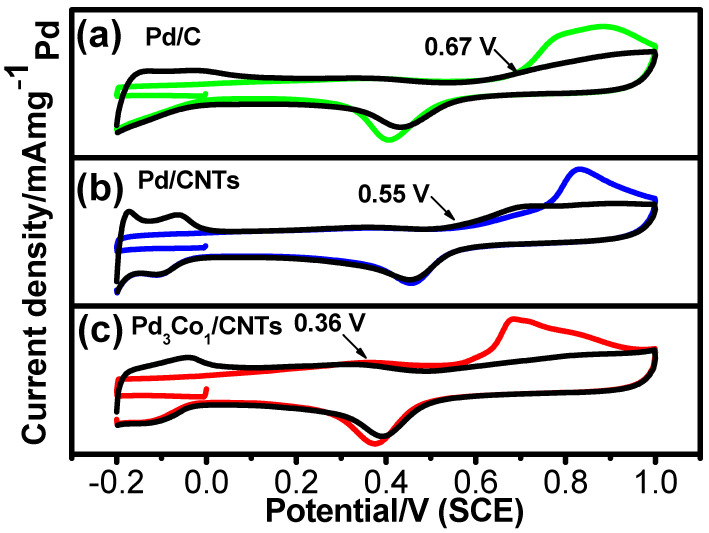
CO stripping voltammograms of Pd/C, Pd/CNT and Pd_3_Co_1_/CNT catalysts in 0.5 M H_2_SO_4_ solution.

We used CO stripping experiments to investigate the CO toxicity of all catalysts. Figure 5 shows the CO stripping voltammograms and the subsequent CV curves. The hydrogen adsorption/desorption were completely suppressed in the low potential region due to the CO_ads_ species on the active sites of the catalysts [59]. Following the removal of CO_ads_, the peaks associated with hydrogen adsorption/desorption reappeared. The onset potential of the adsorbed CO oxidation of Pd_3_Co_1_/CNTs was negatively shifted to 0.36 V, and the corresponding potentials were 0.55 and 0.67 V for Pd/CNTs and Pd/C, indicating that Pd_3_Co_1_/CNTs have an excellent CO oxidation ability.

In conclusion, compared with Pd/C, Pd/CNTs, Pd_1_Co_1_/CNTs and Pd_1_Co_3_/CNTs, Pd_3_Co_1_/CNTs exhibited 1.38-, 1.92-, 1.91- and 2.94-fold enhancement in mass activity toward the FAOR, respectively. The onset potential of the adsorbed CO oxidation of Pd_3_Co_1_/CNTs was negatively shifted to 0.36 V and the corresponding potentials were 0.55, and 0.67 V for Pd/CNTs, and Pd/C. Furthermore, after 7200 s, the Pd_3_Co_1_/CNT catalyst still maintained a higher current density (95.1 mA mg_Pd_^−1^) that was 2.6-, and 29.7- times greater than Pd/CNTs (36.3 mA mg_Pd_^−1^) and Pd/C (3.2 mA mg_Pd_^−1^). The excellent catalytic performance and stability of Pd_3_Co_1_/CNTs can be attributed to the bi-functional mechanism and the obtained Pd_3_Co_1_ alloy nanoclusters. In the bifunctional mechanism, Pd is responsible for the adsorption and oxidative dehydrogenation of formic acid and Co provides the adsorbed hydroxyl group (OH_ads_) at a more negative potential, in comparison with Pd to oxidize the intermediate [22,60]. Moreover, the special Pd_3_Co_1_ alloy nanoclusters can provide abundant catalytic active sites, reduce the charge-transfer impedance, improving the efficiency of the material transfer and further improve the catalytic activity and stability. Furthermore, the Pd_3_Co_1_/CNT catalyst presents the better FAOR mass activity, in comparison with the recent research works on Pd-based bimetallic catalysts [22,26,57,61,62,63,64,65,66,67] (Table S3: Supporting Information).

## 4. Conclusions

Herein, with the assistance of DES, the obtained Pd_3_Co_1_/CNT catalyst exhibited the excellent electrocatalytic performance towards the FAOR. The doping of Co atoms changed the electron configuration of Pd to form a new PdCo bond, thus affecting the adsorption of the toxic intermediates and weakening the dissolution of Pd, which were the important reasons for improving the stability and anti-CO toxicity. Furthermore, these doped Co atoms provided more co-catalytic active sites to obtain more OH^−^ from the H_2_O molecule, which could further interact with the toxic intermediates generated on the Pd active sites to further improve performance. This reaserch provides a new strategy to obtain the Pd-alloy high-performance catalysts for DFAFC.

## Data Availability

Data are available upon request; please contact Pingping Yang, yangpingping0218@163.com.

## Supplementary Materials

The following supporting information can be downloaded at: https://www.mdpi.com/article/10.3390/nano12234182/s1, Figure S1. XRD patterns of Pd_1_Co_1_/CNT (a) and Pd_1_Co_3_/CNTs(b) catalysts; Figure S2. TEM and HRTEM images of the Pd/CNT catalyst; Figure S3: TEM and HRTEM images; HAADF-STEM elements mapping; the corresponding elements of Pd and Co of Pd_3_Co_1_/CNTs (another region); Figure S4. EDX line-profiles (a,b), spot scanning (c,d) of a Pd_3_Co_1_ nanoparticle (where Pd is in red and Co in blue) of Pd_3_Co_1_/CNTs. Figure S5. EDX-spot scanning and element content ratio (b,c) of a Pd_3_Co_1_ nanoparticle in Pd_3_Co_1_/CNTs. Figure S6. The corresponding particle size distribution of a Pd_3_Co_1_/C catalyst. Figure S7. TEM and HRTEM images and the corresponding particle size distribution of Pd_1_Co_1_/CNT (a–c) and Pd_1_Co_3_/CNT (d–f) catalysts. Figure S8. The corresponding particle size distribution of Pd/CNTs. Figure S9. TEM and HRTEM images of a Pd_3_Co_1_/C catalyst. Figure S10 Cyclic voltammograms curve of Pd_3_Co_1_/C in 0.5 M H_2_SO_4_ + 1.0 M HCOOH. Table S1: Elemental composition of the samples obtained from ICP; Table S2: Pd 3d peaks of Pd_3_Co_1_/CNTs and Pd/CNTs; Table S3: A recent literature survey of the activity of the FAOR electrocatalysts.
