# Supplementary material for: Pd3Co1 Alloy Nanocluster on the MWCNT Catalyst for Efficient Formic Acid Electro-Oxidation
Source: Nanomaterials (Basel). 2022 Nov 25;12(23):4182. doi: 10.3390/nano12234182 (PMC9740167; doi:10.3390/nano12234182)
Supplement: Supplementary file 1 [file nanomaterials-12-04182-s001.zip › nanomaterials-2040256-supplementary.pdf]

Supporting Information

Pd_3Co_1 Alloy Nanocluster on the MWCNT Catalyst for Efficient Formic Acid Electro-Oxidation

Pingping Yang †, Li Zhang †, Xuejiao Wei *, Shiming Dong and Yuejun Ouyang *

College of Chemistry and Materials Engineering, Huaihua University, Huaihua 418008, China
* Correspondence: wei1348137@163.com (X.W.); oyyj0816@163.com (Y.O.)
† These authors contributed equally to the work.

Table of Contents:

1. Supplementary Figures

Figure S1. XRD patterns of Pd_1Co_1/CNTs (a) and Pd_1Co_3/CNTs (b) catalysts.

Figure S2. TEM and HRTEM images of Pd/CNTs catalyst.

Figure S3 TEM and HRTEM images; HAADF-STEM elements mapping; the corresponding elements Pd and Co of Pd_3Co_1/CNTs (another region).

Figure S4. EDX line-profiles (a-b), spot scanning (c-d) of a Pd_3Co_1 nanoparticle (where Pd is in red and Co in blue) of Pd_3Co_1/CNTs.

Figure S5. EDX-spot scanning and element content ratio (b-c) of a Pd_3Co_1 nanoparticle in Pd_3Co_1/CNTs.

Figure S6. The corresponding particle size distribution of Pd_3Co_1/C catalyst.

Figure S7. TEM and HRTEM images and the corresponding particle size distribution of Pd_1Co_1/CNTs (a-c) and Pd_1Co_3/CNTs (d-f) catalysts.

Figure S8. The corresponding particle size distribution of Pd/CNTs.

Figure S9. TEM and HRTEM images of Pd_3Co_1/C catalyst.

Figure S10 Cyclic voltammograms curve of Pd_3Co_1/C in 0.5 M H_2SO_4+1.0 M HCOOH.

2. Supplementary Tables

Table S1. Elemental composition of the samples obtained from ICP.

Table S2. Pd 3d peaks of Pd_3Co_1/CNTs and Pd/CNTs.

Table S3 A recent literatures survey of the activity of FAOR electrocatalysts.

3. Supplementary References

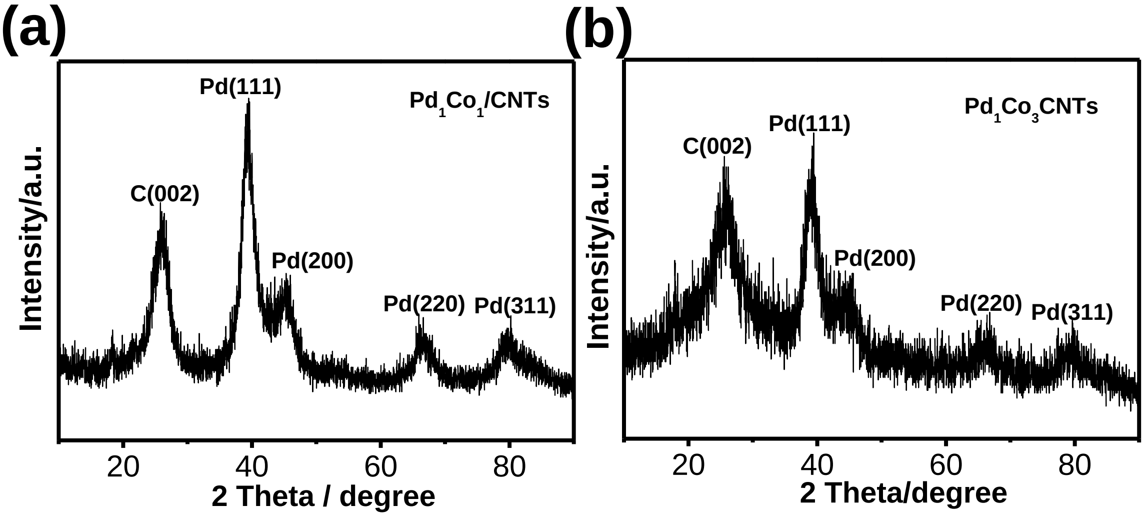

Figure S1. XRD patterns of Pd_1Co_1/CNTs (a) and Pd_1Co_3/CNTs (b) catalysts.

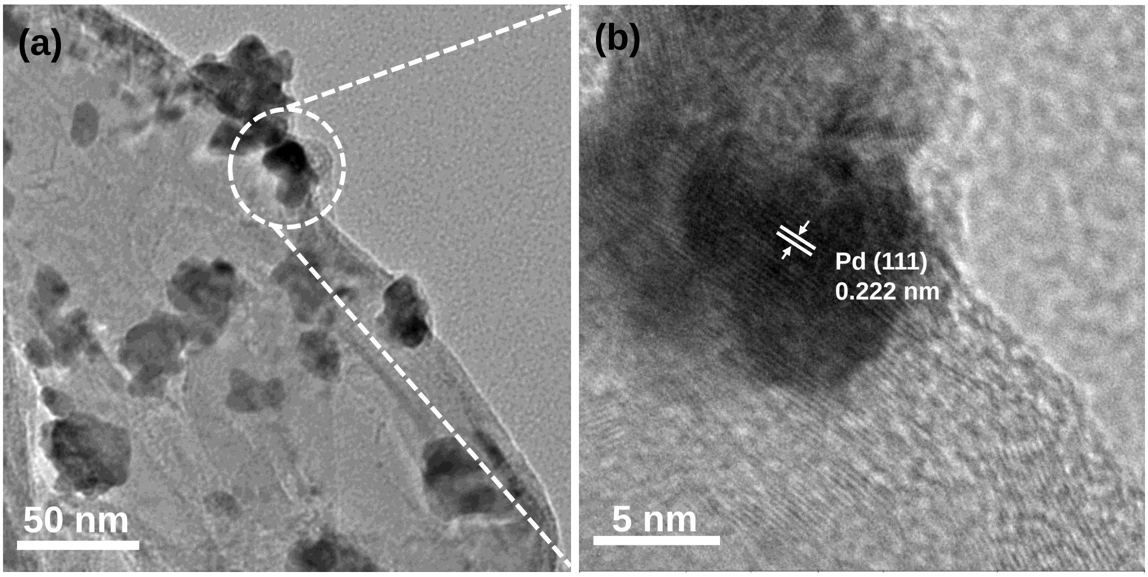

Figure S2. TEM and HRTEM images of Pd/CNTs catalyst.

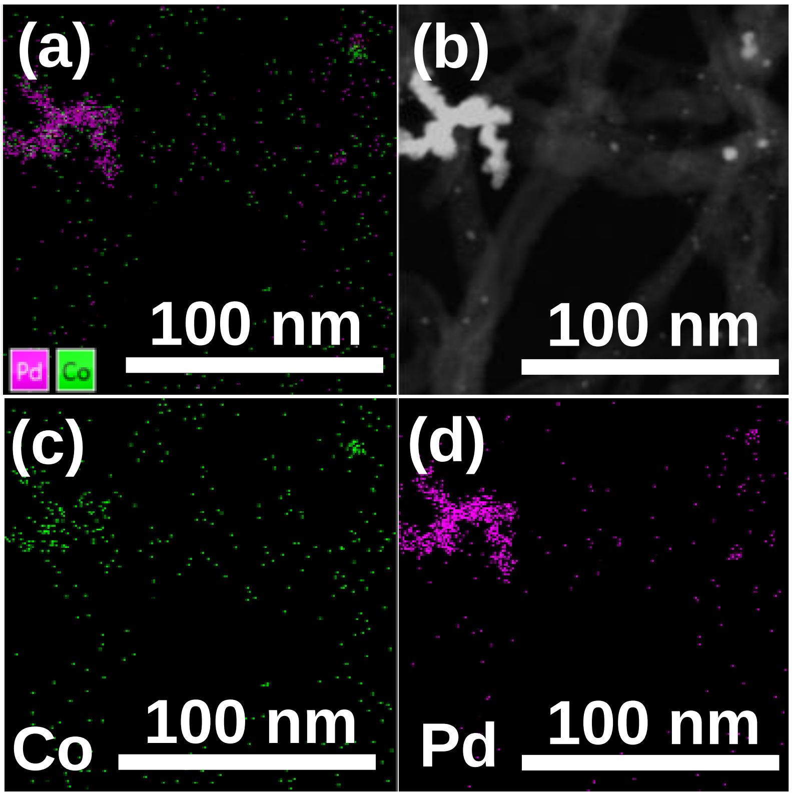

Figure S3. TEM and HRTEM images; HAADF-STEM elements mapping; the corresponding elements Pd and Co of Pd_3Co_1/CNTs (another region).

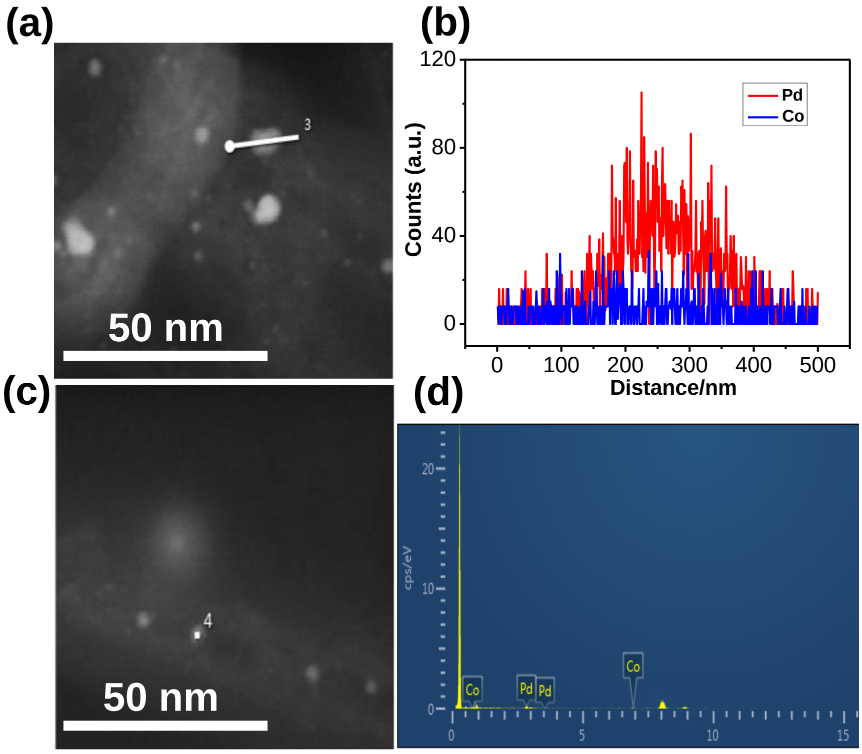

Figure S4. EDX line-profiles (a-b), spot scanning (c-d) of a Pd_3Co_1 nanoparticle

(where Pd is in red and Co in blue) of Pd_3Co_1/CNTs.

(a)

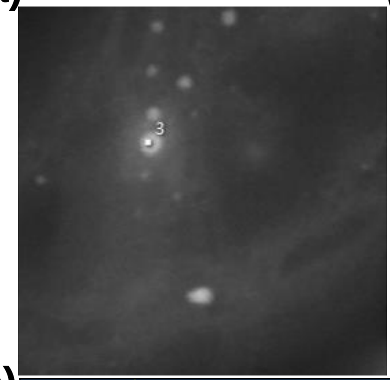

(b)

Element	Line Type	k factor	Absorption Correction	Wt%	Wt% Sigma
Co	K series	1.26119	1.00	15.30	15.30
Pd	L series	1.76392	1.00	84.70	84.70
Total:				100.00	

(c)

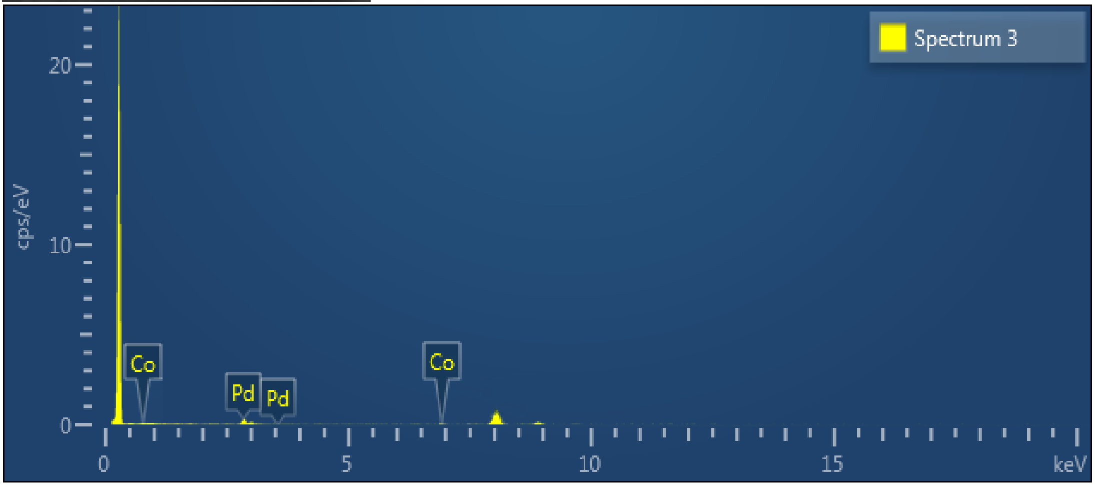

Figure S5. EDX-spot scanning (a) and element content ratio (b-c) of a Pd_3Co_1 nanoparticle in Pd_3Co_1/CNTs.

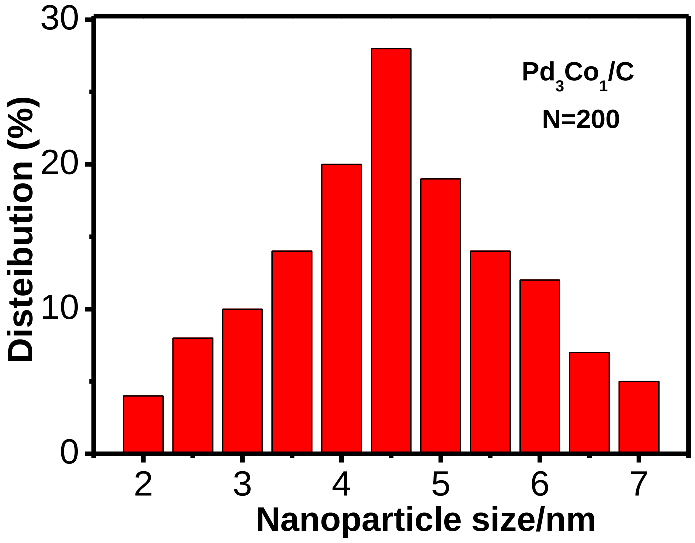

Figure S6. The corresponding particle size distribution of Pd_3Co_1/C catalyst.

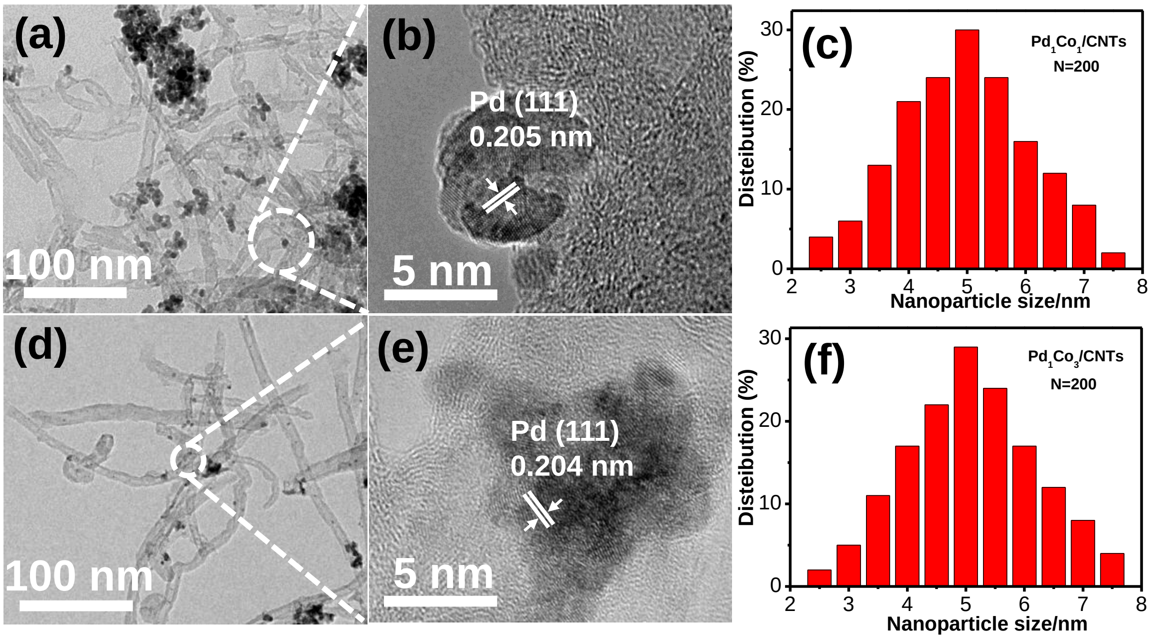

Figure S7. TEM and HRTEM images and the corresponding particle size distribution of $Pd_1Co_1/CNTs$ (a-c) and $Pd_1Co_3/CNTs$ (d-f) catalysts.

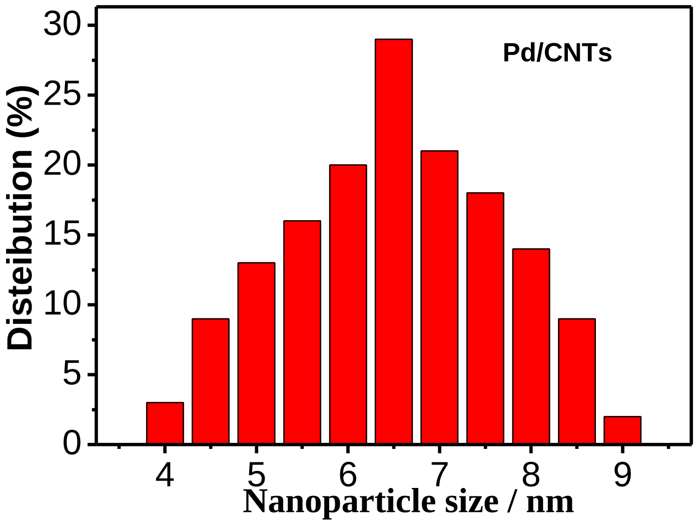

Figure S8. The corresponding particle size distribution of Pd/CNTs.

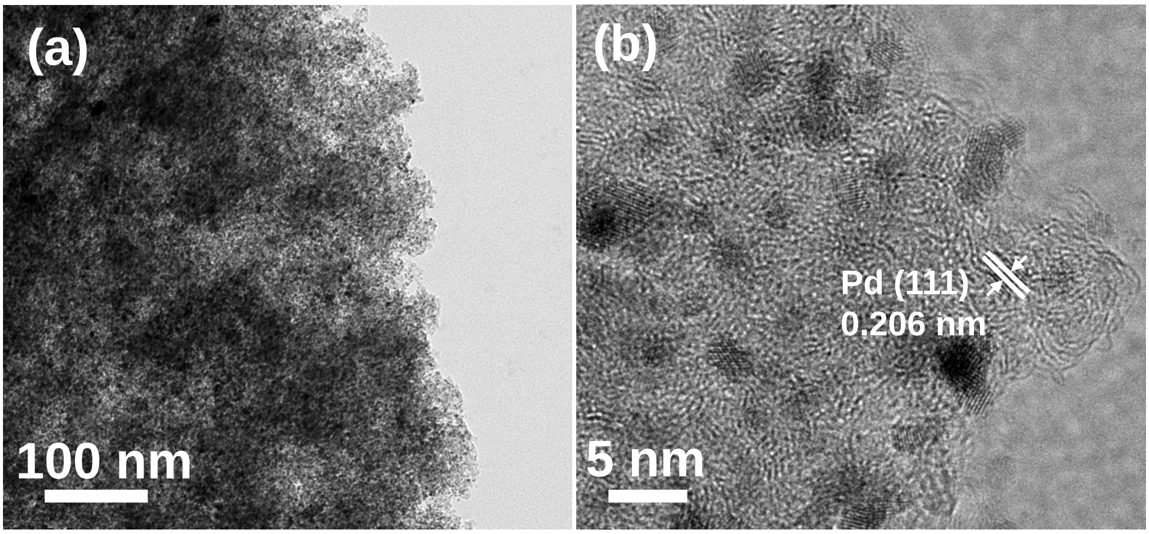

Figure S9. TEM and HRTEM images of Pd_3Co_1/C catalyst.

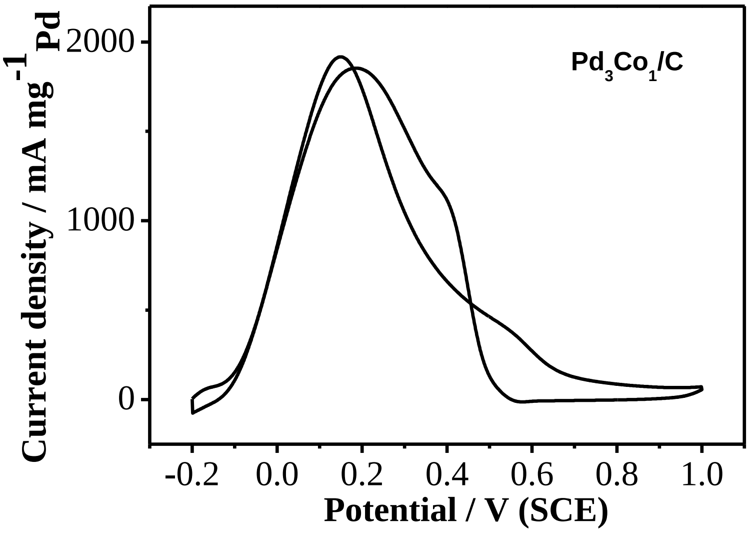

Figure S10 Cyclic voltammograms curve of Pd_3Co_1/C in 0.5 M H_2SO_4+1.0 M HCOOH.

Table S1. Elemental composition of the samples obtained from ICP.

Elements / Catalysts	Pd(wt.%)	Co(wt.%)	Atomic ratios
Pd_3Co_1/CNTs	19.1	6.35	3:1
Pd_1Co_1/CNTs	18.3	17.9	1:1
Pd_1Co_3/CNTs	18.6	55.1	1:3
Pd/CNTs	17.5	-	-

Table S2. Pd 3d peaks of Pd_3Co_1/CNTs and Pd/CNTs.

Catalysts	Pd^0 $3d_{5/2}$ (eV)	Pd^0 $3d_{3/2}$ (eV)	Pd^{2+} $3d_{5/2}$ (eV)	Pd^{2+} $3d_{3/2}$ (eV)
Pd_3Co_1/CNTs	335.8	336.8	340.7	341.3
Pd/CNTs	335.5	336.2	340.6	341.1

Table S3 a recent literatures survey of the activity of FAOR electrocatalysts.

Catalysts	Mass activity ($mA\ mg^{-1}{}_{Pt}$)	References
**Pd_3Co_1/CNTs**	**2410.1**	**This work**
PdCo nanodots	1362.1	[22]
PdCu nanochains	1108.2	[26]
PdCu clusters	1289.0	[57]
coral-like PdCu	1050.0	[61]
Pd_6Co nanocrystals	430.8	[62]
PdCu nanoparticles	194.5	[63]
(3D) porous PdSn	553.4	[64]
PdCu porous network	517.0	[65]
Pd-Fe nanoparticles	1000.0	[66]
PdCu/CNTs	252.0	[67]
